# Physiological monitoring to prevent diving disorders

**DOI:** 10.3389/fphys.2024.1517361

**Published:** 2024-12-18

**Authors:** Paul Beatty, William Evans, Sara Gravelyn, Marshall Tumperi, Druso Daubon, Austin Veith

**Affiliations:** The Johns Hopkins University Applied Physics Laboratory, Laurel, MD, United States

**Keywords:** biometric monitoring, diving disorders, physiological metrics, wearable sensors, diving mishap analysis

## Abstract

Insight into human physiology is key to maintaining diver safety in underwater operational environments. Numerous hazardous physiological phenomena can occur during the descent, the time at depth, the ascent, and the hours after a dive that can have enduring consequences. While safety measures and strict adherence to dive protocols make these events uncommon, diving disorders still occur, often with insufficient understanding of the factors that triggered the event. This review first examines the most common diving disorders and their incidence rates across recreational and US military dive activities. The review then identifies physiological biomarkers (e.g., heart rate, heart rate variability, blood pressure, respiration rate, temperature, oxygen saturation) that may provide a holistic view of the diver’s current physiological state and potentially detect the most concerning diving disorders (e.g., decompression illnesses, gas mixture-related disorders, barotraumas, and environment exposure). Although considerable research is still needed to verify the use of these biometrics in the diving environment, the research described in this review presents a promising path to developing a system that can detect pending diving disorders and provide divers and other necessary parties with an early warning before mishaps occur.

## 1 Introduction

The human body can undergo many aberrant changes during diving activities, which can have devastating long-term consequences. These diving disorders impact both physical and cognitive functioning. They may include complications caused by decompression, breathing gas mixtures at higher partial pressures, equipment malfunctions, not adhering to dive profiles, environmental exposure, and other events discussed in this review. Critically, monitoring human physiology while diving could provide insight into the triggers of these diving disorders, enable the prediction of the onset of dangerous and potentially fatal disorders, and subsequently notify the diver, the diving buddy, and supervisors. However, the environmental constraints of diving such as hyperbaric pressures (Mallen and Roberts, 2019), variable, hypothermic temperatures (Looney et al., 2019), and exposure to conductive, corrosive seawater (Levett and Millar, 2008) have limited sensor development for underwater environments. Additionally, different dive rigs have unique form, fit, and functional constraints that can further limit the use and integration of sensors (Clark, 2015). Despite these challenges, there is a clear need for a dive-able system to collect and store accurate, quantitative physiological metrics that can be relayed in near real-time from the divers to support and supervisory personnel. This review aims to provide an overview of diving physiology, diving disorders, and candidate sensing modalities that could inform the development of underwater physiological monitoring systems.

An understanding of diving physics is necessary to appreciate the complexities of diving disorders. The water pressure on a diver drives the majority of underwater diving disorders. For every 33 feet of seawater (fsw) that a diver descends, the environmental pressure increases by approximately 1 atm or 14.7 psi (Bove, 2014; Bosco et al., 2018). The increase in external pressure causes dramatic changes to lung function, including reductions in vital capacity, residual volume, and lung distensibility, as well as increases in the partial pressures of oxygen and nitrogen (Bosco et al., 2018; Doolette and Mitchell, 2010). For context, the partial pressures of the gases in air at the surface are approximately 0.21 atm absolute (ATA) O_2_ and 0.79 ATA N_2_. At 100 feet deep (approximately 3 atm of additional pressure), the partial pressure rises to 0.84 ATA O_2_, and 3.16 ATA N_2_, respectively (U.S. Department of Commerce, 2024). The greater partial pressures of these gases during a dive result in greater quantities of these gases in blood and tissue. The longer a diver breathes gases at high partial pressures, the more the gases are absorbed. As the gas load increases, several issues begin to emerge.

Two main factors determine the array of possible diving disorders experienced during or after a dive (U.S. Department of Commerce, 2024; Naval Sea Systems Command, 2016; Edge and Wilmshurst, 2021). The first factor, the diving rig (or diving modality), determines the gas mixture and partial pressure of each gas inhaled. Each diving modality provides a mixture of different gasses under increased pressure, and each has a distinct depth limitation. The second factor, the dive profile, includes the time spent at each depth, the temperature of the water, as well as the type of work that the diver is performing. The dive profiles can include a variety of diving activities ranging from short, shallow recreational dives to diving long distances with high levels of exertion (such as in military use cases). Each dive profile presents the diver with unique challenges that, in combination with the breathable gas mixture, provide the basis for diving disorders. For instance, wearing a standard air, self-contained underwater breathing apparatus (SCUBA), a diver performing a deep dive at 130 fsw may be concerned with the progression of nitrogen narcosis, due to the increased nitrogen partial pressure in the gas mixture. However, for a diver in the same scenario but breathing a helium-oxygen (HeO_2_) gas mixture, the progression of nitrogen narcosis is not a matter of concern because the gas mixture contains helium to offset the presence of nitrogen. As another example, military or occupational divers may be exposed to cold water temperatures for extended periods, which are then followed by high stress and high exertion periods at very shallow depths. These divers will be subjected to an increased risk for decompression sickness (DCS) and hypothermic effects due to the cold water environment (Naval Sea Systems Command, 2016).

Diving history is full of serious injuries and fatalities, and many diving disorders were originally identified through empirical evidence and post-mortem analysis (Acott, 1999). Learning from these experiences, the primary mitigation tactic employed by divers has been and remains to be strict adherence to dive profiles. From the earliest work of JS Haldane (Boycott et al., 1908) and the original US Navy dive tables (Acott, 1999) to the advanced algorithms and dive computers utilized today (Azzopardi and Sayer, 2010), the employment of a descent and ascent paradigm to manage nitrogen uptake and off-gas has played an important role in improving diver safety. Specifically, limitations on descent and ascent rates, operating depth, and time spent at depth have been implemented to prevent these issues from arising. These parameters are captured in dive tables as well as the U.S. Navy Dive Manual, which is an evidence-based document for those performing strenuous dives (Naval Sea Systems Command, 2016). While effective, dive tables (depth vs. time) present a non-specific, universal solution to the problem. Even with the dive tables, incidents still occur (see Naval Sea Systems Command, 2016 for dive tables and Section 2 for incidence rate analysis). To detect and prevent these disorders, advances in underwater diver monitoring are necessary (Bube et al., 2022). Knowledge of a diver’s basic vital signs and the onset of diving disorders during and after the execution of their dive is critical for divers, their dive buddies, and dive supervisors. With a more holistic understanding of a diver’s physiological status, tailored dive profiles could be implemented to ensure diver safety and prevent diving disorders before they arise.

Avoidance of diving disorders is critical to diver health and safety, but divers currently lack near real-time physiological and health status monitoring capabilities in many extreme environments (Vinetti et al., 2020). There is an opportunity to leverage sensors developed for air and space over the past few decades to reduce the frequency and severity of diving disorders. Wearable devices are now capable of sensing the environment, basic biometrics (e.g., vital signs), and even complex human performance parameters (e.g., fatigue state, stress response, recovery, and sleep quality). However, this development has primarily taken place on land under normal atmospheric pressure. For instance, electrocardiograms (ECG), which measure voltage potentials on the skin to assess heart function, are easily obtained in dry environments. Yet, due to the conductive nature of seawater, this technology has failed to transition to underwater operational environments and requires more development and validation (Ji et al., 2020; Reyes et al., 2014). The implementation of sensors to notify the diver and their dive buddy would have benefits during basic training, as well as high-risk underwater tasks (e.g., search and rescue).

## 2 Diving disorder incidence rates

To understand which physiological parameters can inform a diver’s current physical and cognitive state underwater, one must first understand how the disorders occur in the first place. Diving fatalities generally follow a linear paradigm, starting with a trigger that leads to a disabling agent, a resulting injury, and an eventual cause of death (Denoble et al., 2008a; Lippmann et al., 2017). For example, dives on air mediums to depths greater than 130 fsw can lead to the onset of nitrogen narcosis (trigger), which can be followed by an uncontrolled ascent due to the mental impairment (disabling agent), which can ultimately result in DCS or an arterial gas embolism (AGE) (disabling injury or diving disorder), which, in extreme cases, can lead to drowning (cause of death). To complicate things further, different diving disorders have disparate consequences that can be more severe than others. The outcome and diver survivability for a diving disorder can vary depending on the affected organ system, the speed of the response, and the available diving medical capabilities.

**FIGURE 1:**
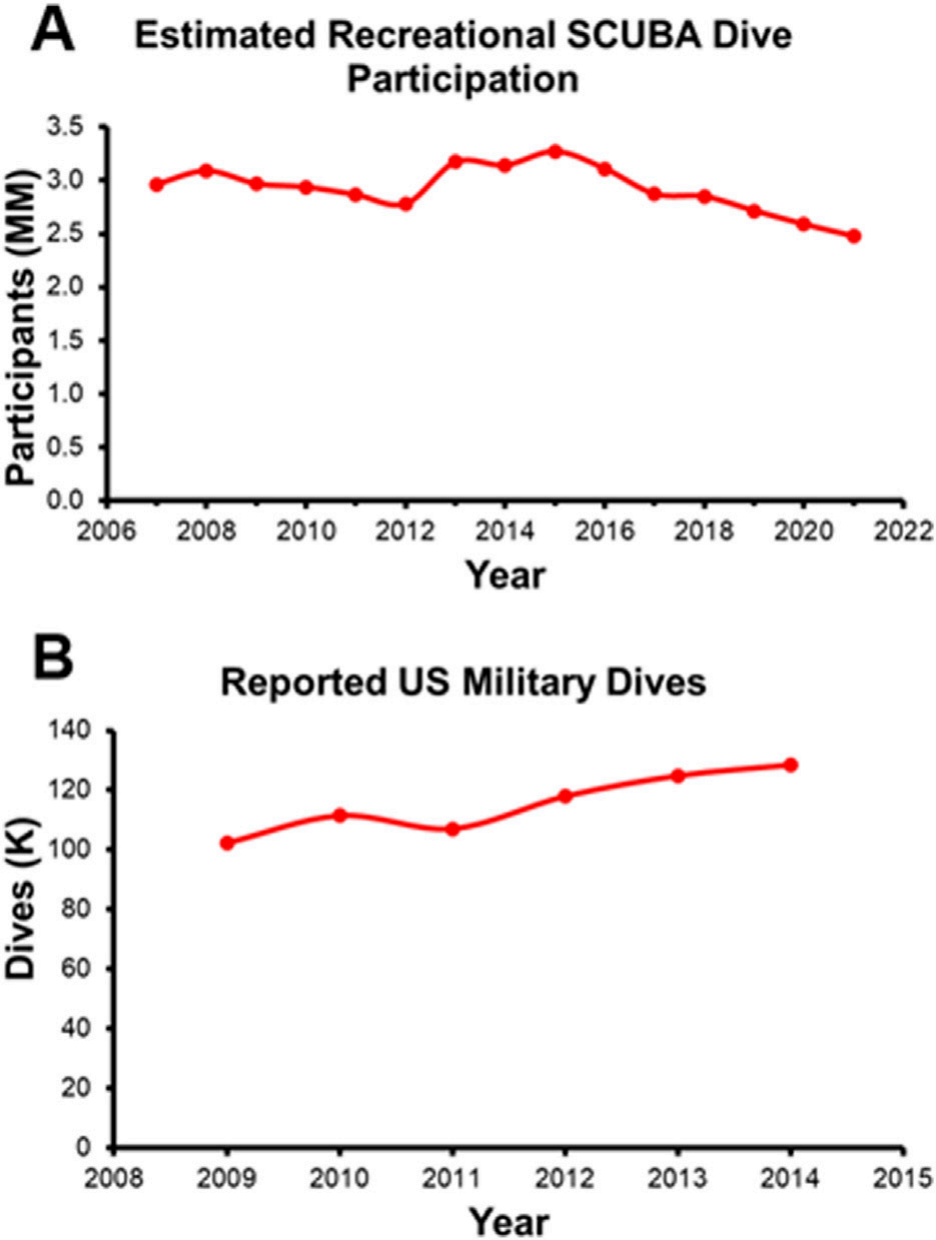
**(A)** Estimated Recreational SCUBA Dive Participation recreated from the 2022 Outdoor Foundation Report. **(B)** Annual Military Wide Dives recreated from Colgary 2016.

To identify the most pertinent disabling events and diving disorders, it is important to observe diving disorder incidence rates. This analysis of diving disorders incidence rates considers recreational diving separately from U.S. Navy military or technical diving due to differences in the participation rate, the demographics of the divers, and the employment of different dive tables and dive profiles. According to the 2022 Outdoor Foundation report, from 2007 to 2021 an average of 2.9 million Americans per year participated in at least one recreational SCUBA dive (recreated in Figure 1A) (Outdoor Industry Association, 2022). In contrast, there were an estimated 100,000–130,000 dives total conducted by the US Navy each year (between 2009 and 2014; Figure 1B) (Colgary, 2016). The two groups are also different in terms of demographic composition, with two notable studies indicating the mean age of a recreational diver being 50.2 (Ranapurwala et al., 2018) compared to the mean age of a Navy diver being 35 at the end of their career (Maguire et al., 2022). Lastly, the dive tables and dive profiles that the two groups followed are distinctly different. While the Navy dive tables were designed for divers who were going to a specific depth for a specific amount of time (Taitt, 2003), the recreational dive community largely adopted more conservative forms of the US Navy tables, due in part to the multi-depth, multi-dive nature of most recreational excursions (Lang and Lehner, 2000). For these reasons, dive disorder incidence rates are explored independently below.

### 2.1 Recreational diving incidence rates

**FIGURE 2:**
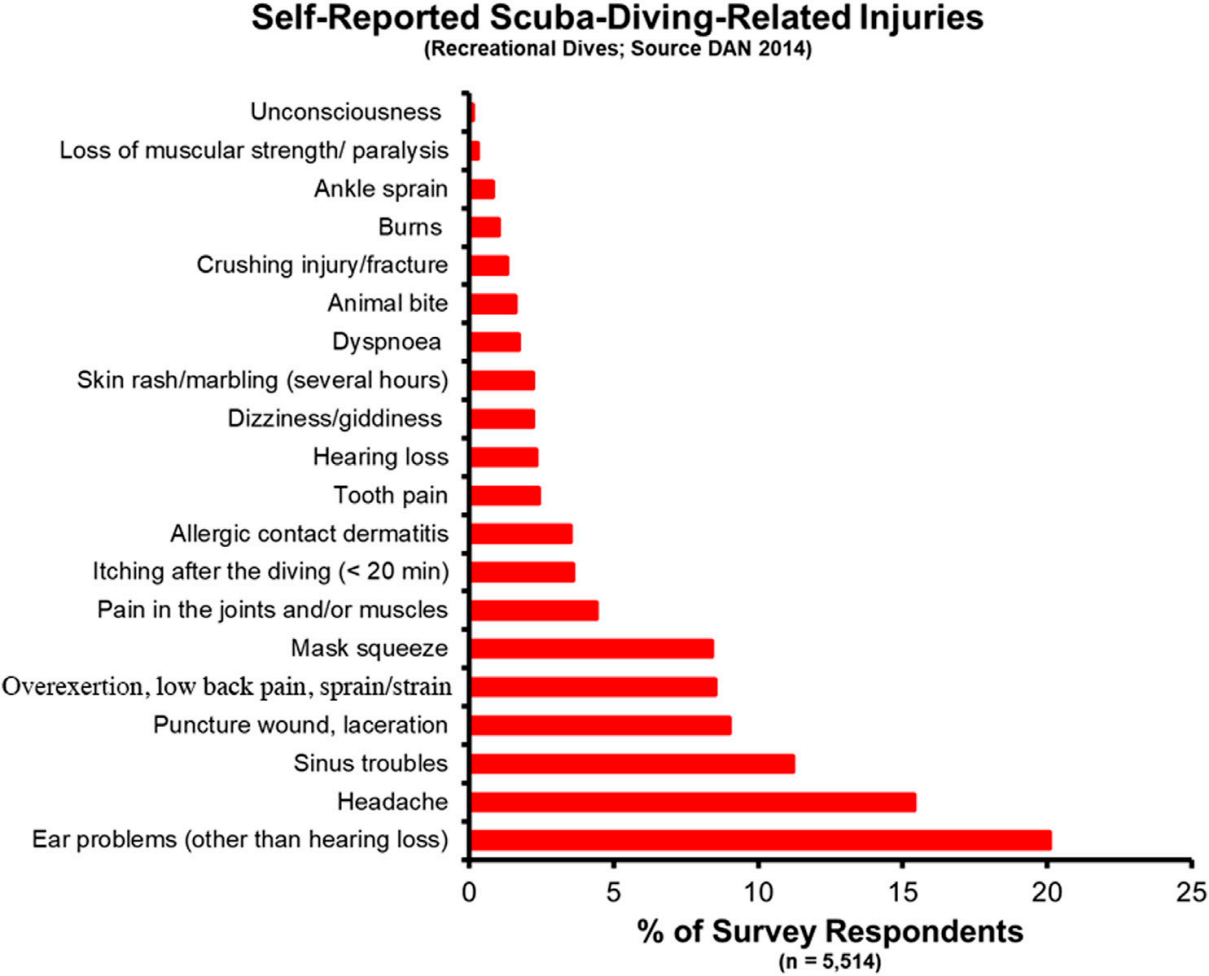
Self-reported SCUBA Diving injuries from a 2014 DAN report.

In the recreational dive world, knowledge of the incidence rate for dive disorders comes from several disparate sources, including the Divers Alert Network (DAN) and other regional authorities. A study of self-reported diving injuries from 5,514 DAN members in 2014 found an overall rate of diving-related injuries of 3.02 in 100 dives (recreated in Figure 2) (Ranapurwala et al., 2014). When broken out by biological sex, males reported an injury rate of 2.65 per 100 dives versus 4.30 per 100 dives for female respondents. 5.8% of respondents reported one of the associated DCS and DCS-related symptoms (pain in the joints and/or muscles, skin rash/marbling, and loss of muscular strength/paralysis). Eleven divers received a total of 18 recommendations for hyperbaric oxygen therapy. The highest reported injury was ear pain, likely associated with the squeeze experienced when a diver fails to clear their ears before changing depths. Other notable findings include a higher incidence rate for all injuries in 17- to 24-year-olds (10.21 per 100 dives vs. 6.75 per 100 dives for 35- to 44-year-olds), an increased injury rate in divers that were overweight and obese [based on Body Mass Index (BMI)], and a decreased injury rate for novice vs. advanced divers. The self-reported data from the DAN study does not include the occurrence of dive fatalities, which is important to understand the incidence of dive disorders along the full scope of severity.

Incidence of recreational dive fatalities can be pieced together by surveying data from DAN (Denoble et al., 2008a; Denoble et al., 2008b), the Professional Association of Diving Instructors (PADI) (Richardson, 2010), and case studies from other regional sources [including Denmark (Vinkel et al., 2016), Australia (Lippmann and McD Taylor, 2020; Lippmann et al., 2013), New Zealand (Lippmann et al., 2021; McClelland, 2007), and others (Casadesús et al., 2019; Ramnefjell et al., 2012; Koca et al., 2018; Ihama et al., 2008)]. As with other self-reported data sets, the incidence rate is likely underreported, but this data serves as a floor for subsequent analysis. One study of the years between 2000 and 2006 showed annual fatality rates varied between 12.1 and 22.9 per 100,000 persons insured by DAN (Denoble et al., 2008b) (average 16.4 per 100,000). By comparison, when examining the fatality rate of PADI instructors, over 10 years (1999–2008), deaths occurred at a rate of only 1.1 per 100,000 members (Richardson, 2010). DAN offers the most comprehensive data on the cause of death and injury in recreational dive fatalities. Annual reports published by DAN between 2004 and 2018 report the most common disabling injuries and causes of death, respectively, are drowning (16% and 40%), cardiac events (19% and 13%), and AGE (10% and 7%) (Tables 1, 2) (Divers Alert Network, 2007; Divers Alert Network, 2006; Divers Alert Network, 2008; Pollock et al., 2009; Pollock et al., 2010; Pollock et al., 2011; Trout et al., 2015; P Buzzacott, 2016; P Buzzacott, 2017; Buzzacott, 2019; PJ Denoble, 2019; F Tillmans, 2020). DAN studies published before 2010 found similar results, with two notable differences (Denoble et al., 2008a; Denoble et al., 2008b). Cardiac arrest, AGE, and drowning comprised 26%, 17%, and 24% of the cause of death, respectively. A higher incidence of AGE in the literature is consistent with higher rates of AGE fatalities reported in DAN annual reports during this period as compared to recent years. A 2008 study examining recreational open-circuit scuba diving deaths from 1992 to 2003 found the most common disabling injuries were asphyxia (33%), AGE (29%), and cardiac incidents (26%) (Denoble et al., 2008a). Notably, there are only 8 deaths (all in 2004) and 2 disabling injuries (1 in 2007 and 1 in 2017) attributed to asphyxia in the 15 years of DAN reports. These documents indicate equivalence between asphyxia and drowning in reporting. Thus, these cases have been included under “Drowning/Probable drowning” categories in Tables 1 and 2. Similar to injury incidences, multiple studies have identified a higher percentage of dive fatalities occurring in divers with greater than normal BMI (Lippmann et al., 2021; Casadesús et al., 2019), tracking with the higher injury incidence rates seen in previous studies. In these cases as well, asphyxia (or drowning), AGE, and cardiac incidences were the most common disabling injuries.

**TABLE 1:** Disabling injury for US and Canada recreational dive fatalities 2004–2018 (source: DAN).

	2018	2017	2016	2015	2014	2013	2012	2011	2010	2009	2008	2007	2006	2005	2004	Total
Drowning/Probable drowning	6	1	7	6	13	13	13	15	14	8	10	38	22	—	25	**191**
Cardiovascular/Cardiac event	12	9	6	9	21	8	16	25	13	27	28	26	13	—	12	**225**
Arterial Gas Embolism (AGE)	6	1	0	6	1	2	4	4	19	12	13	13	15	—	20	**116**
Decompression Sickness (DCS)	0	1	3	0	0	0	1	3	0	2	1	1	1	—	0	**13**
Immersion Pulmonary Edema	6	0	0	0	0	2	2	6	0	0	0	0	0	—	0	**16**
Loss of consciousness	3	1	7	1	1	3	1	3	1	0	0	0	0	—	2	**23**
Trauma	0	0	0	0	1	0	0	0	0	0	0	4	0	—	5	**10**
Seizure	0	0	0	0	2	0	2	1	0	2	1	2	1	—	0	**11**
Breathing gas intoxication	0	0	0	0	0	0	1	0	0	0	0	0	0	—	3	**4**
Other^a^	1	3	0	1	2	0	2	3	5	0	0	0	1	—	1	**19**
Unknown	21	54	71	44	27	48	39	38	20	29	30	22	22	89	20	**574**
**Total**	**55**	**70**	**94**	**67**	**68**	**76**	**81**	**98**	**72**	**80**	**83**	**106**	**75**	**89**	**88**	**1,202**

^a^ Other includes Alcohol and medication intoxication, Gastrointestinal bleeding, Hypothermia, Hypoxia, Lung overexpansion, Respiratory distress, Shark bite, Water aspiration.

Bold values indicate the sum of values for the respective row and column in the table.

**TABLE 2:** Cause of death for US and Canada recreational dive fatalities 2004–2018 (source: DAN).

	2018	2017	2016	2015	2014	2013	2012	2011	2010	2009	2008	2007	2006	2005	2004	Total
Drowning/Probable drowning	15	11	20	14	24	21	28	26	36	34	35	68	50	36	56	**474**
Cardiovascular/Cardiac event	4	5	9	11	16	8	13	23	5	8	9	14	5	10	10	**150**
Arterial Gas Embolism (AGE)	2	2	0	5	2	2	2	4	14	11	12	8	2	5	9	**80**
Decompression Sickness (DCS)	0	1	3	0	0	0	1	4	1	1	1	1	1	0	0	**14**
Immersion Pulmonary Edema	2	0	0	0	1	2	2	7	1	0	0	0	0	0	0	**15**
Other^a^	2	0	0	0	1	1	3	2	2	0	0	0	0	1	4	**16**
Unknown	30	51	62	37	24	42	32	32	9	24	26	15	17	37	9	**447**
**Total**	**55**	**70**	**94**	**67**	**68**	**76**	**81**	**98**	**68**	**80**	**83**	**106**	**75**	**89**	**88**	**1,198**

^a^ Other includes: Anoxic brain injury, Carbon monoxide poisoning, Gunshot wound to the head, Medication intoxication, Multi-organ failure, Pulmonary barotrauma, Shark bite, Trauma.

Bold values indicate the sum of values for the respective row and column in the table.

### 2.2 Military diving incidence rates

On the military side, every diving mishap and near mishap is reported to the US Navy Safety Command (NSC) (Colgary, 2016; O’Connor et al., 2007). These reports include hazardous behaviors and near misses to permanent disabilities and fatalities, and include all Department of the Navy (DON) divers, including the US Marine Corps divers. The data on military diving incidence rates are sparse and, like the recreational diving world, must be pulled from multiple sources (O’Connor et al., 2007; Jóźwiak et al., 2015; Walsh et al., 2024; Turner et al., 2024). Two specific citations are worthy of in-depth reporting here. The first report by O’Connor et al. (2007) examined US Navy mishaps between 1993 and 2002 (O’Connor et al., 2007). This analysis of 263 US Navy diving mishaps showed that the most common dive injury reported was DCS I/II (122 cases), followed by AGE (97 cases). The other incidents reported in O’Connor et al. (2007) included oxygen toxicity (5 cases), death caused by drowning or trauma (5 cases), and near drowning (5 cases). The remaining 29 cases included mechanical injuries, chemical injuries, and various asymptomatic missed decompression instances. It is worth noting that a similar report between 1968 through 1981 indicated that 41.1% of all mishaps could be attributed to DCS (Hoiberg et al., 1985), tracking with the relative percentage between 1993 and 2002.

**FIGURE 3:**
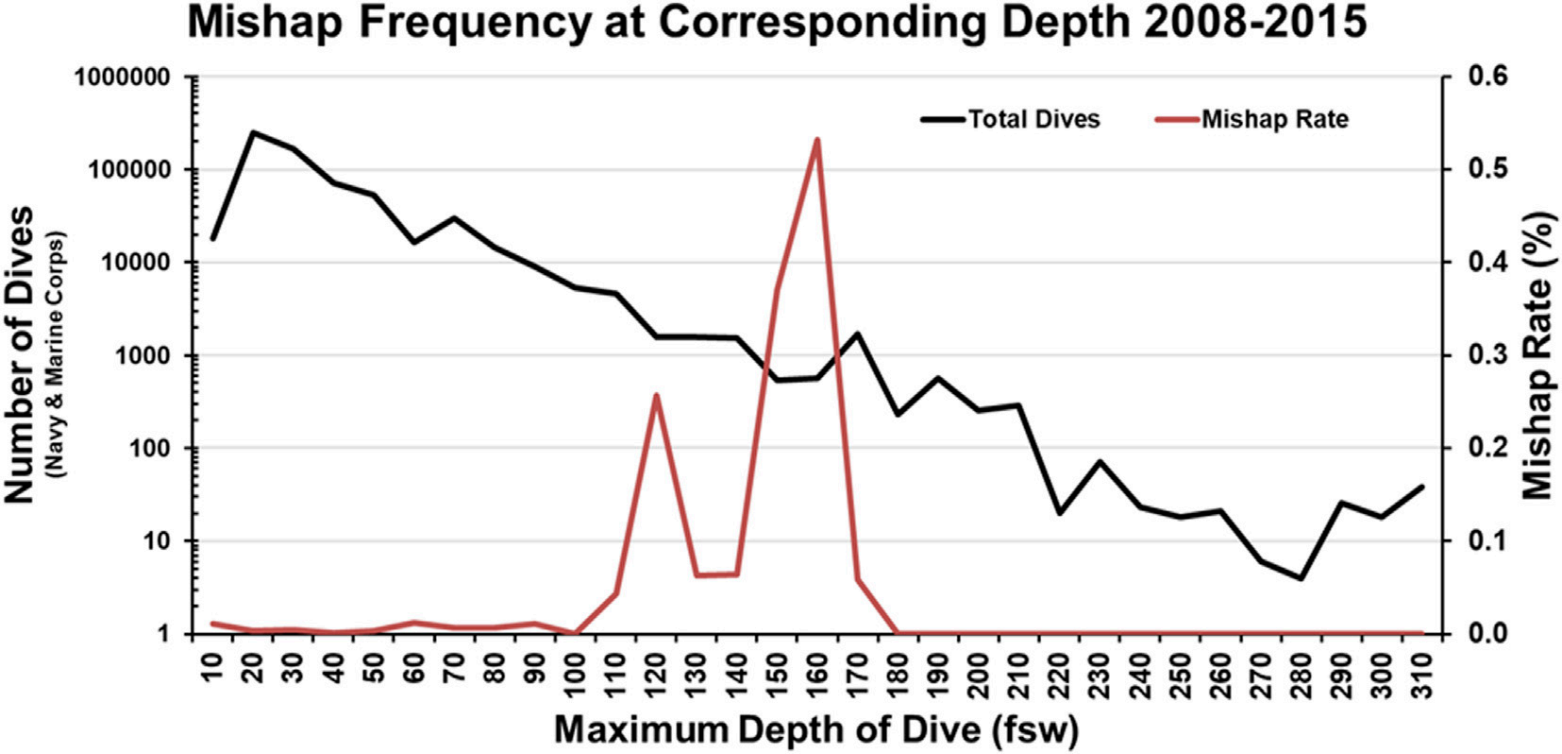
Mishap Frequency at Corresponding Depth 2008–2015 recreated from Colgary 2016.

The second report of note on military diving injury rates comes from Colgary 2016 (Colgary, 2016). In the summer of 2015, a Freedom of Information Act application was approved, turning over 768,851 dive log entries from the US NSC, including 39 mishap reports from 2008 to 2015 and 343 additional mishap reports from 1960 to 2007. The first part of the analysis concerned the frequency and depth (binned in 10 fsw increments) of the military dives reported. Most depth bins were dominated by training (both student and supervisor), with 57% of all dives less than 190 ft constituting training dives. Of note, 90% of all military dives contained in the dataset were 60 ft or less with a median dive depth of 20 ft. Pressure testing (60 ft), recompression treatments (60 ft), and requalification dives (10–19 ft) drove an appreciable component of this effect. In terms of dive apparatus, SCUBA (51.8% of all dives), surface supplied (19.9%) and oxygen rebreathers (19.2%) constituted the three most common modalities. Finally, there were an average of 115,000 dives a year, predominately executed by the Navy. The second half of the analysis laid out in Colgary 2016 concerns the mishap reports. The mishaps contained in the dataset averaged less than 10 per year and were less than 0.01% when compared to all dives. However, when the dive frequency and dive depth are taken into consideration, this rate rises to a maximum of 0.5% in some depth bins (Figure 3). This low incidence rate was attributed to the high level of training, supervision, and technical capability. However, mishaps still warrant investigation and mitigation, as they have the potential to cause lasting and life-threatening effects. Some of the more common mishaps mirror the recreational world, with AGE, DCS, and barotraumas occurring up and down the water column (Table 3).

**TABLE 3:** Total Number of Navy Diving Incidents Recorded from 2008 to 2015 from Colgary et al., 2016.

Physiological issue	Incidents recorded
Arterial Gas Embolism	16
Barotrauma	4
Chemical Injury	2
DCS Type I/II	4
Hypercapnia	3
Drowning/Near Drowning	4
Other	8
**Total**	**41**

Bold values indicate the sum of values for the respective row and column in the table.

## 3 Diving disorders and sensing technologies

The following section describes each of the diving disorders in greater detail. Table 4 and the text in Section 3 couple the diving disorders to a measurable physiological parameter that could be incorporated into a device or other diving system. These relevant physiological metrics include heart rate (HR), heart rate variability (HRV), core temperature, blood pressure (BP), oxygen saturation (peripheral: SpO_2_, muscle: SmO_2_, and tissue: StO_2_), and respiration rate (RR).

**TABLE 4:** Potentially relevant metrics for detecting common diving disorders^a^.

Item	Disorder	Description	Biometrics	System metrics
Decompression Illnesses				
1	Gas Embolisms	Obstruction of blood flow caused by gas bubbles (emboli) in the vasculature	HR, BP	Pressure (Environmental)
2	Decompression Sickness	Excess gas separates from solution and forms bubbles in the bloodstream	HR, HRV	Pressure (Environmental)
Gas Mixture-Related Disorders				
3	Hypoxia	Deficiency of oxygen in the arterial blood	HR, HRV, BP, RR, SpO_2_, SmO_2_, StO_2_	O_2_ (Rig), CO_2_ (Rig)
4	Oxygen Toxicity (CNS)	A collection of primarily neurological symptoms resulting from exposure to oxygen partial pressures exceeding 1.3 ATA (wet) or 2.4 ATA (dry)	HR, HRV, Electrodermal	Pressure (Environmental) O_2_ (Rig)
5	Hypercapnia	Abnormally high levels of carbon dioxide in the blood and body tissues	HR, SpO_2_, RR	O_2_ (Rig), CO_2_ (Rig)
6	Nitrogen Narcosis	A state of euphoria that occurs when a diver breathes a gas mixture with a nitrogen partial pressure greater than approximately 4 ATA	EEG, CFFF	Pressure (Environmental), Pressure (Rig)
7	Chemical Injury	Introduction of a caustic solution from the carbon dioxide scrubber of the UBA into the upper airway of a diver (“Caustic Cocktail”)	HR, BP, RR Core Temperature Skin Temperature	Humidity Sensor (Rig)
8	CO Poisoning	A buildup of carbon monoxide in the bloodstream	HR, RR	Pressure (Environmental) CO (Rig)
Barotraumas				
9	Ear Barotrauma “The Squeeze”	Damage to body tissues from the mechanical effects of pressure Includes: Middle and inner ear, nasal sinus	—	Pressure (Environmental)
10	Subcutaneous Emphysema	Air is forced into the loose body tissues and trapped under the skin	—	—
11	Pulmonary Overinflation	A lung overexpansion injury caused by inadequate exhalation, especially during rapid ascent	—	—
12	Pneumothorax	Air is trapped in the pleural space between the lung and the chest wall	HR, RR, SpO_2_	Pressure (Rig)
Environment Exposure				
13	Hypothermia	Lowering of the core temperature of the body	Core Temperature, Skin Temperature	Ambient Temperature
14	Hyperthermia	Raising of the core temperature of the body	Core Temperature, Skin Temperature	Ambient Temperature
15	Immersion Pulmonary Edema	A condition that causes infiltration of fluid from the bloodstream into the alveoli	—	—
Other Diving Disorders				
16	Asphyxia (Drowning)	A condition where breathing stops and both hypoxia and hypercapnia occur simultaneously	HR, SpO_2_, RR, Physical Activity	—
17	Involuntary Hyperventilation	Breathing more than is necessary	RR, Physical Activity	Pressure (Rig)

^a^ List is non-exhaustive; Additionally, a number of these techniques are still experimental and not fully operationalized.

### 3.1 Decompression illnesses (AGE, VGE, DCS)

Collectively known as decompression illnesses, arterial gas embolisms (AGE), venous gas embolisms (VGE), and decompression sickness (DCS), are common diving disorders that occur due to the formation of gas bubbles in the vasculature when there is a reduction in environmental pressure during ascent (Vann et al., 2011; Walker et al., 2023; Mitchell et al., 2022). The main distinction between these disorders is the mechanism by which they occur. While an AGE occurs when a gas bubble in the arterial bloodstream blocks the blood supply to an organ (resulting in symptoms resembling a stroke or tissue infarct), a VGE occurs when gas inside of tissues that are discharged into the venous bloodstream expands and prevents the gas from exiting the body (Neuman, 2002). The ineffective elimination of gas bubbles from the body (specifically in a diving decompression context) is what leads to the variety of negative symptoms collectively referred to as DCS (colloquially known as “the bends”) (Neuman, 2002). DCS can be further delineated into type I (which involves symptoms only related to the skin, musculoskeletal system, or lymphatic system) and type II (which includes symptoms from type I with additional symptoms related to nervous system function) (Liew and Flynn, 2005). Critically, although gas bubbles can cause AGE if they travel through a patient’s foramen ovale in the heart (Moon, 2019), not all bubbles will produce DCS symptoms (Nishi, 1972). Most bubbles will be eliminated by the lungs without deleterious effect (Moon, 2019). DCS typically occurs due to procedural error (e.g., the diver stayed underwater for too long or ascended too quickly). The risk of DCS has been shown to increase on extended-duration dives (Naval Sea Systems Command, 2016), and it is thought that colder dives have a greater risk of DCS than warmer dives (Gaustad et al., 2021). However, other evidence suggests that more research is required to fully understand the impact of ambient temperature and decompression risk. A recent body of research has also challenged the traditional DCS pathophysiology, indicating that DCS symptoms may be the result of a complex inflammatory response (Gaustad et al., 2021; Arya et al., 2023; Bigley et al., 2008; Leffler, 2001; Toner and Ball, 2004). Nevertheless, additional research is necessary to cement the validity of this hypothesis and develop operationalized diagnostic tools.

Although it is difficult to differentiate gas embolisms from decompression sickness (Mitchell et al., 2022), treatment of these disorders typically follows the same protocol: Divers who lose consciousness during ascent (or very shortly afterward) are promptly treated with 100% oxygen and recompression, albeit with mixed efficacy (Bessereau et al., 2010; Blanc et al., 2002; Sen and Sen, 2021). Symptoms associated with arterial occlusion (i.e., seizure, cardiac arrhythmia, coronary infarction, etc.) can provide a differential diagnosis (Alexander and Martin, 2023); however, further research is necessary to fuse physiological signals, such as HR and BP, into a meaningful diagnostic picture. Despite their current shortcomings in gas embolism identification, the use of cardiovascular measurements has the potential to assist in the diagnosis of DCS. More specifically, research has shown that time, depth, and gas mixture affect HR and HRV (Noh et al., 2018). In a small sample, dry-suit divers wearing a portable ECG demonstrated depressed HR measurements at depth as well as changes in both HRV time and frequency domain characteristics. Further, results from Bai et al. (2009) and Bai et al. (2013) both presented preclinical DCS models in swine that indicated DCS development both elevates (Bai et al., 2013) and depresses (Bai et al., 2009) parasympathetic system activity (Bai et al., 2013). This effect manifested in differential ECG morphology, specifically an increased T-wave amplitude (Bai et al., 2013), likely stemming from DCS-related acidosis, but the effects on markers like HRV remain in question. Additional research from Schirato et al., 2020 has also shown a relationship between decompression-induced physiological stress and HRV, this time in humans. Interestingly, the authors commented on contradictory results presented by Bai et al., suggesting they were likely due to an experimental design error, and Schirato et al. went on to demonstrate changes in both time and frequency domain HRV measurements, in particular the standard deviation of normal R to R (NN) intervals (SDNN), that were observed after decompression. However, Schirato et al. also stressed the unstable nature of HRV measurements, noting a large variation in the measurement among and within subjects. Taken together, there is likely some relationship between DCS and cardiovascular function; however, more research is needed before diagnostic indicators could be employed in a system for divers.

The US Navy defines an acceptable risk of DCS to be 2% for mild cases (type I DCS) and 0.1% for serious cases (type II DCS) (Liew and Flynn, 2005). An analysis of Navy dives between 1971 and 1978 showed an overall DCS rate of approximately 1.3%, with some dive schedules reaching a 4.8% DCS incidence rate (Berghage and Durman, 1980). It should be noted that from 1957 to 2007, the dive tables for standard air decompression remained relatively unchanged. In 2008, the US Navy Diving Manual (Revision 6) updated the decompression tables for nitrox gas, with the major change requiring longer decompression stops over the 1957 tables (Gerth and Doolette, 2009). While this resulted in a lower incidence of decompression sickness, the US Navy still defined the severity of the DCS to be unacceptable, with all cases resulting in central nervous system (CNS) involvement (DCS type II) (Doolette et al., 2009). The most current revision to the tables (Revision 7) has further reduced the risk of DCS to less than 3%; however, evidence shows the severity of the VGEs remains high (Andrew and Doolette, 2020). One of the major issues with any decompression table is its uniform approach. Several factors increase the personal risk of DCS, including water temperature, work rate, immersion diuresis, and pre-existing conditions. With advances in bloodstream bubble detection, technologies such as ultrasound may be able to sense and predict gas embolisms (Karimpour et al., 2022; Eftedal et al., 2007; Papadopoulou et al., 2018). While there are some technologies that monitor divers post-dive, to our knowledge, there are no waterproof technologies that detect bubbles in real time on a diver, at depth (Currens et al., 2024; Doolette, 2016). Additionally, more research is needed to strengthen the link between bubble formation and detection and decompression sickness diagnosis (Papadopoulou et al., 2013; Eckenhoff et al., 1990).

### 3.2 Gas mixture-related disorders

Several diving disorders can occur due to nuances in the consumption of compressed gas mixtures in underwater, hyperbaric environments. This section briefly discusses common diving disorders related to insufficient oxygen (hypoxia), excess oxygen (hyperoxia) and its downstream consequences, excess carbon dioxide (hypercapnia), excess nitrogen (nitrogen narcosis), or other disorders related to the gas mixtures (such as chemical injuries or CO poisoning). Although these disorders are discussed in the same section due to their similar mechanism of injury, it is critical to understand that these disorders should not be conceptualized in the same manner for diver safety. Moreover, these disorders can be distinguished in terms of the frequency by which they occur, as well as the severity of the consequences should they occur.

#### 3.2.1 Hypoxia

Hypoxia is an abnormal deficiency of oxygen in the arterial blood in which the partial pressure of oxygen is too low to meet the metabolic needs of the body (Wiebe et al., 1999). An environment is considered hypoxic when the partial pressure of oxygen in the breathing gas is below 0.16 ATA, regardless of depth. The onset of hypoxic effects can be sudden and can arise without warning. Additionally, hypoxia is a common cause of injury and fatality in semi or closed-circuit breathing rigs (Popa et al., 2022). On the surface, as the closed-circuit diver consumes oxygen, the oxygen fraction in the breathing loop will begin to decrease, as will the gas volume in the breathing bag. Oxygen is typically added on demand into the breathing loop. If the valves malfunction, no oxygen will be added and the oxygen fraction may drop to ten percent or less, instantiating hypoxic breathing conditions. Since the partial pressure of oxygen is constantly increasing on descent, hypoxia is less likely to occur while divers are descending. However, the reverse is also true. Hypoxia can be accelerated by the sudden decrease in oxygen partial pressure due to ascent.

The relevant biometric to measure hypoxia is SpO_2_. SpO_2_ is a proxy measurement of the percent of oxygenated hemoglobin in the arterial blood (Nitzan et al., 2014). SpO_2_ measurements above 95% are considered healthy and values below 90% are generally considered hypoxic (American Thoracic SocietyAmerican College of Chest Physicians, 2003). Acute hypoxia, even acute profound hypoxia (SpO_2_ <70%), for up to 10 min, is generally well tolerated in humans with no long-lasting effects (Bickler et al., 2017). However, increasingly prolonged exposure to hypoxic conditions can lead to progressively severe instances of shallow-water blackout (Bart and Lau, 2022). While ascending, the decreasing partial pressure of oxygen in the breathing loop, combined with the metabolic consumption of oxygen, creates an environment where there is a high demand for oxygen in the body, but oxygen is actively transferred back to the breathing loop. This combination leads to an increased risk of unconsciousness as cerebral oxygenation drops. Other relevant biometrics include SmO_2_ and StO_2_ (Hoiland et al., 2016), RR, and HR. Non-traditional oxygen saturation measurements are still an active area of research and are not as well established as SpO_2_. Alternatively, well-established metrics, like HR and RR, may be particularly relevant as hypoxic conditions signal the cardiac output centers in the brain to increase HR and RR (Zhang et al., 2014; Brinkman et al., 2023). As the sympathetic nervous system affects these changes, it follows that HRV could be a relevant metric. However, the data does not support this relationship during physical activity (Buchheit et al., 2004), and the relationship between HRV and hypoxia (i.e., whether or not changes in HRV could be used to predict these events) warrants further study.

There are several clinical-grade medical devices, as well as commercial off-the-shelf (COTS) products, that can measure SpO_2_ with a high degree of fidelity on land. Some researchers have made strides to adapt this technology to the underwater operating environment (Mulder et al., 2021; McKnight et al., 2021). A key challenge with these devices is their accuracy. The accuracy of pulse oximeters begins to decrease as the oxygenation percentage decreases because they are generally calibrated to a range of saturation between 70% and 100% (Torp et al., 2023). Other sources of inaccuracy include but are not limited to, skin tone, movement, body placement, core temperature, and blood flow (Fine et al., 2021). Similarly, the use of traditional methods such as an ECG to measure HR, presents challenges underwater versus on land. Specifically, ECG signal quality is dramatically impaired, particularly in seawater (Reyes et al., 2014). While there have been examples of ECG successfully measured in a dry suit configuration (Noh et al., 2018), additional developments are necessary to ruggedize ECG devices for all possible deployments. Furthermore, there are practical limitations to the adhesion of ECG pads. A surrogate measure such as pulse rate might be reasonable, but limitations also persist due to reductions in blood flow that occur in cold environments often present during dives (Yeung et al., 2016). Moreover, the pulse rate may misreport HR if an arrhythmia is present, as each heartbeat may not result in changes in blood flow, blood pressure, and pulse rate. Finally, accurate measurement of the gasses (O_2_, CO_2_) could factor into a novel safety system; however, further exploration is needed.

#### 3.2.2 Oxygen toxicity

When a diver breathes oxygen at high partial pressures, this can prompt the diver to become over-oxygenated (hyperoxia), which can subsequently lead to a diving disorder known as oxygen toxicity (O_2_ toxicity) (Cooper et al., 2023). In hyperbaric conditions, the increase in partial pressure of the oxygen in the lungs has several physiological effects, with known and unknown mechanisms (Thomson and Paton, 2014). O_2_ toxicity is understood to be dose-dependent. Both dive depth (pressure) and dive duration can be altered to mitigate the effects of oxygen on the body. There are two main types of O_2_ toxicity: pulmonary (Jackson, 1985) and CNS (Rodgers et al., 2018); the former is better understood than the latter (Naval Sea Systems Command, 2016). Pulmonary O_2_ toxicity likely occurs as the result of free radical oxidation in the pulmonary tissue. Oxidative stress causes lung irritation, with symptoms including chest pain, cough, and pain on inspiration. These symptoms can develop slowly and become increasingly worse as long as elevated oxygen levels are inspired, with prolonged exposure to high concentrations of oxygen leading to pulmonary edema. Pulmonary O_2_ toxicity can occur whenever the partial pressure of oxygen exceeds 0.5 ATA. In place of personal physiological monitoring, established safety factors have successfully mitigated oxygen toxicity. However, monitoring RR may provide utility for detecting oxygen toxicity, as changes in RR often occur downstream of pulmonary O_2_ toxicity (Cooper et al., 2023; Lellouche and L’Her, 2020).

The more insidious form of oxygen toxicity is CNS O_2_ toxicity. High partial pressures of oxygen are associated with many biochemical changes in the brain, but it is unknown which specific changes are responsible for the signs and symptoms of CNS O_2_ toxicity. CNS O_2_ toxicity can occur whenever the oxygen partial pressure exceeds 1.3 ATA in a wet diver or 2.4 ATA in a dry diver. The symptoms of CNS O_2_ toxicity may be remembered with the following acronym, VENTIDC (Naval Sea Systems Command, 2016):

V: Visual symptoms. Tunnel vision, a decrease in the diver’s peripheral vision, and other symptoms, such as blurred vision, may occur.
E: Ear symptoms. Tinnitus, any sound perceived by the ears but not resulting from an external stimulus, may resemble bells ringing, roaring, or a machinery-like pulsing sound.
N: Nausea or spasmodic vomiting. These symptoms may be intermittent.
T: Twitching and tingling symptoms. Any of the small facial muscles, lips, or muscles of the extremities may be affected. These are the most frequent and clearest symptoms.
I: Irritability. Any change in the diver’s mental status including confusion, agitation, and anxiety.
D: Dizziness. Symptoms include clumsiness, incoordination, and unusual fatigue.
C: Convulsions. The first sign of CNS oxygen toxicity may be convulsions that occur with little or no warning.

The exact mechanism of CNS O_2_ toxicity is unknown; however, it is hypothesized that reactive oxygen species interrupt neural networks and function (Manning, 2016). Initial studies have demonstrated that electrodermal activity can predict CNS O_2_ toxicity in divers (Posada-Quintero et al., 2021). However, this measure has not yet been ruggedized and implemented in a technology suitable for the underwater operating environment. Although HRV could be relevant due to its utility as a measure of sympathetic outflow, research is lacking in this area and the inclusion of HRV would be an exploratory biometric. Reductions in HR stroke volume and cardiac output have been shown in patients suffering from hyperoxia (Rodgers et al., 2018); however, it is not understood how this translates to an underwater environment.

#### 3.2.3 Hypercapnia

Hypercapnia is a condition that is either due to hypo-ventilation or the inhalation of a gas mixture with a substantial fraction of carbon dioxide (CO_2_). More specifically, a build-up of CO_2_ in the breathing supply will accumulate in the diver’s blood and tissues (Dunworth et al., 2017), which can lead to symptoms such as headaches, mild dizziness, and confusion (Sterba, 2024). In high quantities, this excess CO_2_ can cause cognitive/physiological impairment and ultimately lead to diving accidents. The principal causes presented in the US Navy Dive Manual are the following:

1) Excess carbon dioxide levels in compressed air supplies due to improper placement of the compressor inlet.
2) Inadequate ventilation of surface-supplied helmets or UBAs.
3) Failure of CO_2_ absorbent canisters to absorb CO_2_ or incorrect installation of breathing hoses in closed or semi-closed circuit UBAs.
4) Low lung ventilation relative to exercise level

Notably, inadequate lung ventilation is often caused by skip breathing, increased apparatus dead space, excessive breathing resistance, or increased oxygen partial pressure (Dunworth et al., 2017). To mitigate instances of hypercapnia, specific procedures for filling, packing, and weighing the CO_2_ absorbent material are followed. Additionally, diving profiles and water temperatures are meticulously calculated and factored into operational planning. These steps are taken to ensure diver safety, not just to prevent hypercapnia, but also CNS O_2_ toxicity. It is hypothesized that hypercapnia greatly increases the risk of CNS O_2_ toxicity by increasing brain blood flow and consequently brain oxygen levels, leading to toxic concentrations of oxygen in the brain (Eynan et al., 2020). CO_2_ sensors in the breathing apparatus in conjunction with SpO_2_ monitors could potentially help mitigate hypercapnia events. Additionally, insight into the diver’s RR (i.e., rapid increases or decreases) could help identify hypercapnic breathing patterns (Brinkman et al., 2023). In contrast to hypoxia, hypercapnia decreases RR, triggering a pattern of slow, deep breaths, as the lungs aim to limit dead space and optimize CO_2_ clearance. Finally, HR may also be relevant, as a rise in CO_2_ in the bloodstream initially depresses HR due to an increasing intercellular pH (van Beek et al., 1998).

#### 3.2.4 Nitrogen narcosis/inert gas narcosis

Nitrogen narcosis is a condition that temporarily impairs the neurological function of a diver, resulting from the inhalation of high concentrations/partial pressures of nitrogen in the breathing apparatus (Rocco et al., 2019). Similar to oxygen toxicity, as the diver descends, the partial pressure of nitrogen increases, leading to an increased concentration of nitrogen in the bloodstream. While the exact mechanism is unknown, a leading hypothesis suggests that there is a correlation between the lipid solubility of an inert gas and its narcotic effect (Kirkland et al., 2022). Therefore, at higher concentrations, more gas is dissolved in the lipid bilayer of cells, leading to greater interaction with cellular processes and disrupted cell signaling (Hapfelmeier et al., 2000). As a diver descends to greater depths, the probability of experiencing narcotic effects from the gases that interfere with the diver’s nervous system increases, potentially leading to a significant impairment in their judgment or adherence to protocol. Nitrogen narcosis effects are progressive, as an increase in the diver’s depth can lead to complete incapacitation if not recognized promptly. Measurements of electrical activity in the central nervous system (electroencephalography; EEG) or measurements of other autonomic processes could be leading indicators of a narcotic event (Dolmierski et al., 1988; Pastena et al., 2005). Although nitrogen narcosis can be evaluated at depth during a dive using the critical flicker fusion frequency (CFFF; see Section 4), other methods have been employed to prevent nitrogen narcosis, such as the utilization of other gas mixes (helium-oxygen; heliox), which eliminates the narcotic effects of nitrogen, especially at deeper depths. Helium, unlike other inert gases, does not have narcotic properties when inhaled at high partial pressures.

#### 3.2.5 Chemical injury

Chemical injury is often referred to as a “caustic cocktail” and can have immediate adverse effects on a diver (Minns et al., 2010). This injury is caused by excessive humidity mixing with a carbon dioxide absorbent (Sodasorb; soda lime) to create a caustic alkaline solution in the breathing loop of a closed-circuit diving apparatus. If inhaled, the solution can cause corrosive esophageal and lung injuries. Choking, gagging, foul taste, and burning of the mouth and throat may begin immediately (Buzzacott et al., 2022). Although the onset of a “caustic cocktail” in itself does not cause a diving physiological disorder, the exposure to this solution underwater may cause a diver to immediately ascend to the surface, which could be the impetus for an AGE, barotraumas, a hypoxia event, or DCS, depending on the diving modality. If this caustic solution reaches the upper airway of the diver, it can cause several adverse effects including hyperventilation, headache, and with prolonged exposure, significant injury to the pulmonary system (Minns et al., 2010).

The extent of the chemical injury depends on the amount and distribution of the solution. Additionally, the accumulation of the caustic solution in the canister can impair the carbon dioxide absorption properties of the system, leading to other issues like hypoxia and hypercapnia. Advancements in breathing loop sensing technologies could lead to diver warnings that could alert the diver ahead of a chemical injury; however, no known interventions have been operationalized. Poisonings and other chemical ingestions have been shown to alter some biometrics (abnormal heart rates, body temperatures, blood pressures, etc.) (Lee et al., 2008; Gorguner and Akgun, 2010), and therefore the sudden inhalation of a strong alkaline solution would likely cause some detectable change in a diver’s vital signs. However, there is no research showing a causal relationship between any biometric data to these events.

#### 3.2.6 Carbon monoxide (CO) poisoning

Carbon monoxide (CO) is a colorless, odorless, tasteless gas that, when inhaled, will ultimately bind to hemoglobin present in red blood cells and thus prevent oxygen from being circulated throughout the body (Long and Flaherty, 2017). The inability to adequately oxygenate the body (tissue hypoxia) due to the binding of CO is referred to as CO poisoning. Despite being an uncommon diving hazard (Hampson, 2020), CO poisoning can be very dangerous. One of the most common causes of CO poisoning in a diving context is procedural error when charging air cylinders or nitrogen and air gas mixes used in Nitrox diving. Accordingly, closed-circuit UBAs that only employ oxygen are not rigs involved with CO poisoning. Charging these cylinders in the presence of other contaminants can contaminate the pressurized gas. Although the presence of small amounts of CO can be unnoticeable at low pressures, the effects of CO poisoning worsen as the gas is present at higher partial pressures. CO poisoning can be particularly detrimental to divers, as it can be confused with decompression illnesses such as DCS (Allen, 1992) or AGE (Holt and Weaver, 2012). Treatment of CO poisoning is similar to the treatment of decompression illnesses, which is to have the diver breathe high concentrations/partial pressures of oxygen in hyperbaric environments (Köhler et al., 2022).

Similar to sensing chemical injuries, advancements in breathing loop sensing technologies could provide a diver with an advanced warning that CO is present in the breathing apparatus. However, given the seemingly infrequent occurrence of CO poisoning, no known interventions for CO poisoning have been operationalized. One physiological device that may be capable of detecting CO poisoning is a pulse CO-oximeter (Hampson, 2012). Analogous to pulse oximeters collecting peripheral oxygen saturation (SpO2), these devices are capable of detecting CO saturation, or more specifically, elevated blood carboxyhemoglobin (COHb) levels. This would be a novel addition to an underwater physiological monitoring system; however, it has been suggested that COHb levels do not correlate well with the clinical severity of CO poisoning (Garg et al., 2018). Critically, elevated COHb levels have also been shown to incorrectly elevate the oxygen saturation from pulse oximetry, which suggests that metrics such as SpO2 may not provide utility for CO poisoning (Hampson, 1998). Nevertheless, other physiological metrics that may help to detect CO poisoning can include HR and RR, as both of these metrics have been well characterized in the literature, albeit in a clinical setting. Analyzing these metrics for an underwater use case will require additional research.

### 3.3 Barotrauma

Barotrauma is an inclusive term for a diverse group of pressure-induced injuries that occur when a gas-filled body space is blocked off from the environment and subsequent pressure equalization occurs due to a change of volume of trapped gas (Hamilton-Farrell and Bhattacharyya, 2004). This results in a range of disparate prognoses, from mild cutaneous bruising to tympanic membrane ruptures, to irreversible tissue damage (Haake et al., 1987). The following section discusses a selection of the most common diving-related barotraumas prioritized as a function of complication severity (from least to most severe).

#### 3.3.1 Ear squeeze

Barotrauma associated with the ear canal, colloquially referred to as “ear squeeze,” (Mawle and Jackson, 2002) is one of the most commonly reported injuries that occur when diving that can vary in severity depending on where trauma occurs. Ear squeezes can occur at depths of as little as eight feet and stem from blockages in the nasal tissue when a diver has a head cold or cannot clear their ear passages (eustachian tube) correctly (Sheridan et al., 1999). While middle ear barotrauma usually resolves on its own, inner ear barotrauma typically requires medical intervention for symptoms related to tinnitus, hearing loss, vertigo, or permanent auditory disability (Sheridan et al., 1999). More specifically, if a person does not ascend to more shallow depths when pain is observed, this can lead to increasingly more severe injuries (i.e., tympanic membrane perforation), which occur suddenly and would require inspection of the ear canal to confirm. Thus, ear squeeze currently lends itself to be exceptionally difficult for physiological monitoring systems to detect and prevent, and currently relies on the diver to discern during descent.

#### 3.3.2 Subcutaneous emphysema

Subcutaneous emphysema is a type of barotrauma that occurs when gases are trapped under the skin and inside body tissues (Aghajanzadeh et al., 2015). Although subcutaneous emphysema is considered to be a common, non-life-threatening diving complication, it can range in severity, appearing as swelling in regions such as the face, neck, upper chest, and shoulders. The presence of subcutaneous emphysema can also be a sign that air is occupying somewhere deeper in the body and can be a symptom of another type of barotrauma known as pneumothorax, which is described later in this review. Treatment of subcutaneous emphysema is dependent on various methods that can be used to release the trapped air such as incisions, needles, or drains (Aghajanzadeh et al., 2015). Non-invasive methods to detect subcutaneous emphysema in real-time currently do not exist.

#### 3.3.3 Pulmonary overinflation

Positive pulmonary barotrauma, or pulmonary overinflation syndrome (POIS), is a lung overexpansion injury that occurs during ascent. It is primarily caused by inadequate exhalation, especially during rapid ascent, as lung volume expands with decreasing environmental pressure (Kennedy-Little and Sharman, 2024). Expansion exceeding the elastic limit of alveoli can lead to rupture, and subsequent release of air (Bove, 2014). In severe cases, alveolar air can enter pulmonary capillaries and central circulation, leading to an AGE. Depending on the rupture location, air may move centrally, entering the mediastinum, or peripherally to the pleural space. Central movement may result in pneumomediastinum or subcutaneous emphysema, if air dissects upwards toward the head and neck. Peripheral movement to the pleural space may result in a pneumothorax or tension pneumothorax (Kennedy-Little and Sharman, 2024). Patients with pneumomediastinum may be asymptomatic, or may experience chest pain, coughing, shortness of breath, or dysphagia. Hamman’s sign, a crunching sound heard during auscultation, is also a symptom of pneumomediastinum. Most mediastinal air reabsorbs on its own and does not require treatment, however breathing 100% oxygen can increase reabsorption rate (Bove, 2014). To our knowledge, there are currently no real-time monitoring devices capable of assessing pulmonary overinflation.

#### 3.3.4 Pneumothorax

A pneumothorax is a condition where air becomes trapped in the pleural space between the lung and the chest wall. It occurs when differences in pressures result in a one-time leakage of air, causing partial lung collapse and varying degrees of respiratory distress (Zarogoulidis et al., 2014). Symptoms of a pneumothorax include sudden, sharp chest pain, shortness of breath, labored breathing, rapid heart rate, weak pulse, and anxiety. In severe cases, a damaged lung may allow gas to enter, but not leave. Successive breaths increase the volume of this gas pocket, in turn increasing pressure on the lung and heart, potentially leading to a tension pneumothorax (Zarogoulidis et al., 2014). If unmitigated, a tension pneumothorax eventually leads to complete lung collapse and consequently, impaired respiration and circulation. A tension pneumothorax will become progressively more severe, eventually leading to shock and death.

The treatment approach depends on the size and severity of the pneumothorax. Smaller pneumothoraxes (less than 15%) may resolve on their own as the trapped air is reabsorbed spontaneously (Naval Sea Systems Command, 2016) and can be managed by having the affected individual breathe 100% O_2_. However, more significant pneumothoraxes (with cardio-respiratory compromise) may require active intervention such as insertion of a chest tube, large-bore intravenous catheter, or other devices designed to remove intrathoracic gas. Patients with pneumothorax may develop tachycardia, tachypnea, and hypoxia, especially as the injury progresses (McKnight and Burns, 2023). As with a number of the other diving disorders, monitoring HR, RR, and pulse oximetry levels could potentially help detect the presence of a pneumothorax (Gustman et al., 1983), however, further research is required to deploy these methods underwater. Other monitoring technologies include ultrasound imaging, which is used clinically to diagnose the presence of pneumothorax, but again has not been outfitted to underwater applications (Alrajhi et al., 2012).

### 3.4 Environment exposure

In addition to diving disorders derived from changes in environmental pressure or imbalances for inspired gas, there are a couple of diving disorders that can occur due to the body’s inability to effectively regulate body temperature in extreme environments. For instance, failure to retain heat in cold environments (hypothermia) or failure to release heat in hot environments (hyperthermia) are two extremes of the temperature regulation continuum for maintaining homeostasis. Additional exposure-related conditions are also discussed.

#### 3.4.1 Hypothermia

On one end of the spectrum, hypothermia is a condition characterized by a drop in the body’s core temperature, which poses a significant risk during diving operations in cool and cold waters. Hypothermia occurs when the body loses more heat than it generates due to the temperature difference between the body and water (Aguilella-Arzo et al., 2003). Critically, the thermal conductivity of water is 26 times greater than the thermal conductivity of air (Sterba, 1989), which suggests that divers may become hypothermic faster underwater. Therefore, a diver’s response to immersion depends on the level of thermal protection worn and the ambient water temperature. Even in relatively warm water, if given enough time, a diver can become hypothermic without an adequate level of protection. Initially, cold-induced vasoconstriction reduces blood flow to the skin, lowering heat conductance and preserving the body’s core temperature (Sterba, 1989). However, this vasoconstrictive regulation has limitations, and prolonged exposure to frigid water can lead to fluctuating blood flow and increased heat loss. Exercise in cold water accelerates heat loss, as movement stirs water in contact with the skin, promoting convection (Kuehn, 1978). For context, an average core temperature is approximately 98.6°F. As a diver’s core temperature drops, symptoms of hypothermia progress from uncontrolled shivering and slurred speech in mild cases (core temperatures between 95°F and 97°F) to loss of shivering, impaired mental status, irregular heartbeat, and shallow respirations in the most severe cases (core temperatures between 88°F and 93°F) (Mccullough and Arora, 2004). A core temperature of 84°F or below generally results in a loss of consciousness (Mccullough and Arora, 2004). Given enough time in cold water, even the most protected divers can experience severe core temperature drops and resultant hypothermic symptoms.

#### 3.4.2 Hyperthermia

At the other end of the spectrum, hyperthermia is a condition characterized by a high core body temperature due to either excessive thermal protection or prolonged exposure to warmer ambient water temperatures (Looney et al., 2019). One of the primary methods that the body uses to reduce core body temperature is to sweat. Moreover, the evaporation of sweat is what aids in the cooling process (Gagnon et al., 2018). However, being immersed underwater prevents this process and hinders a diver’s ability to shed excess heat. When the core body temperature gets too high, a person can experience symptoms such as nausea, slurred speech, headache, vertigo, body weakness, and dehydration. In severe cases, a diver would experience heat fatigue, a drop in blood pressure, and loss of consciousness. To mitigate the occurrence of these diving complications, divers are advised to wear appropriate thermal protection based on water temperature, expected bottom time, and individual factors. Monitoring of ambient water, skin, and core temperatures could help detect these events before they become dangerous. Monitoring skin temperature to predict diving disorders would require extensive algorithm development, as skin temperature can fluctuate across a wide range of temperatures in response to environmental conditions (Yousef et al., 2024). However, some efforts have been made to correlate core body temperature with skin temperature readings (Eggenberger et al., 2018; Xu et al., 2013). Recent developments related to skin surface heat flux sensors, could provide a more ergonomic, less invasive solution to traditional core temperature measurements (Daanen et al., 2023; Ajčević et al., 2022), however more research is needed to validate these sensors at depth and in water.

#### 3.4.3 Immersion pulmonary edema

Immersion pulmonary edema, also referred to as swimming-induced pulmonary edema (SIPE), is a condition that causes infiltration of fluid from the bloodstream into the alveoli (Harper, 2011). Since first being reported in the late 1980s (Wilmshurst et al., 1989), no single pathophysiology adequately describes the occurrence of SIPE. However, multiple intrinsic and extrinsic factors have been proposed which contribute to an individual’s risk (Moon et al., 2016; Peacher et al., 2015; Sames et al., 2019). Chronic hypertension is the greatest predictor for those who experience SIPE, including individuals who develop hypertension later in life. While typically a result of cardiac problems, such as heart failure, SIPE has a high incidence in healthy swimmers during strenuous activity (Shupak et al., 2000; Miller et al., 2010). Additionally, water immersion leads to an increased central blood volume and thus raised cardiac filling pressures (Koehle et al., 2005). In colder water, this effect is further exacerbated due to the vascular response to cold temperatures in addition to immersion. Finally, negative lung breathing pressures, due to use of different breathing apparatus and immersed postures, contribute to hydrostatic pressures driving fluid movement into the alveoli from pulmonary capillaries (Wilmshurst, 2019; Shupak et al., 2003). Each unique risk factor may act to increase the pressure gradient across the pulmonary capillary membrane, which in turn increases the combined likelihood of SIPE. SIPE can be fatal in severe cases, and otherwise presents with symptoms of dyspnea, cough, hemoptysis, and sputum production (Adir et al., 2004). Monitoring of pulmonary capillary pressure would enable the calculation for the risk of developing SIPE, however, this is typically an invasive process involving right heart catheterization (Ranu et al., 2010). Noninvasive measurements have been shown to correlate with pulmonary capillary wedge pressure at normobaric conditions (Nagueh et al., 2019; Genovese et al., 2021), but a dive-ready monitoring method has not been developed. More research is needed to identify correlates to pulmonary capillary pressure or alternate approaches that can be applied in wet, hyperbaric conditions to enable better tracking and understanding of SIPE pathophysiology.

### 3.5 Diving complications - asphyxia and involuntary hyperventilation

Other diving complications worth mentioning relate to changes in the natural breathing dynamics of divers over time. Asphyxia, or suffocation, is a condition where there is a simultaneous lack of oxygen (hypoxia) and excess carbon dioxide (hypercapnia), typically caused by an interruption in breathing. The mechanism for why asphyxia occurs is not completely understood. On the other end of the spectrum, involuntary hyperventilation is a condition in which a diver breathes more than is necessary, presumably due to a sense of anxiety related to a perceived lack of air while underwater. Both hyperventilation and asphyxia can lead to blackouts/syncope underwater and ultimately death of the diver. While monitoring the diver’s HR, SpO2, and RR can provide a means to identify changes in breathing dynamics, it would be difficult to disentangle changes in these metrics from other diving disorders. Changes in accelerometry data or other measurements of physical activity could also provide a differential diagnosis (Zhang et al., 2024), however, development is still required.

## 4 Underwater cognition

In addition to examining diver cardiovascular and pulmonary health, another consideration for designing systems that are capable of real-time physiological monitoring is the immediate, short-term, and perhaps long-term impacts on the nervous system and diver cognition, which are briefly described in this section. It is essential to monitor diver cognition to not only prevent diving mishaps (due to changes in cognitive/behavioral functioning throughout a dive) but also mitigate the occurrence of preventable nervous system damage. Diver cognition has been assessed in a variety of contexts including pre/post dive surveys/testing (Kovacs and Paulsen, 2017; Zec et al., 2023), neurocognitive assessments [in dry environments (Kowalski et al., 2011), combining dry land and underwater testing (Dalecki et al., 1985; Phillips, 1984; Steinberg and Doppelmayr, 2017; Tsai et al., 2020), underwater testing at various depths (Boucher et al., 2022; Möller et al., 2023; Williamson et al., 1989; Hobbs et al., 2014) or hyperbaric conditions (Brookhuis et al., 1994; Lewis and Baddeley, 1981; Lafère et al., 2019; Todnem et al., 1989; Dolmierski et al., 1988; Tikkinen et al., 2016)], psychophysiological testing approaches [electroencephalography (EEG) (Todnem et al., 1989; Dolmierski et al., 1988; Ergen et al., 2017; Todnem et al., 1991), electrodermal activity (Posada-Quintero et al., 2021)], as well as longitudinal neuroimaging [computed tomography (CT) (Slosman et al., 2004) and magnetic resonance imaging (MRI) (Todnem et al., 1991; Balestra and Germonpré, 2016; Coco et al., 2019)]. The following section provides a high-level discussion of the current state of underwater cognitive science.

### 4.1 Neurocognitive assessments

There are a variety of neurocognitive assessments that interpret subtle changes in performance on behavioral paradigms as a method to inform changes in cognitive ability compared to baseline performance. Each test has been empirically shown to assess a specific domain of cognition. For example, a test battery known as the automated neuropsychological assessment metrics (ANAM) was created for the United States Department of Defense (DoD) (Reeves et al., 2007) and is still widely used for assessing military servicemember cognition (Meyers and Vincent, 2020) and in clinical interventions (Woodhouse et al., 2013). It includes tests sensitive to changes in attention, concentration/vigilance, reaction time, memory, processing speed, decision-making, and executive function. Most studies will utilize similar neurocognitive assessments (or subsets of tests) that are present in the ANAM. Critically, neurocognitive assessments rely on longitudinal pre/post evaluation of cognition and therefore produce discrete scores for specific components of cognition. It is possible that taking a battery of tests in an underwater use case may prove to be too cognitively invasive to be incorporated into a real-time monitoring device (i.e., several trials must occur to obtain meaningful results - per cognitive component being assessed).

Nevertheless, neurocognitive assessments have still provided insight into changes in cognition that take place in divers across different environments. Divers often state that they experience a sense of cognitive impairment while at depth. Although there is work exploring potential causal factors [such as inhaled gas (Lafère et al., 2019; Lowe et al., 2007; Grønning and Aarli, 2011), temperature (Lowe et al., 2007; Baddeley et al., 1975; Piispanen et al., 2021; Sharma et al., 2023), pressure (Brookhuis et al., 1994; Lewis and Baddeley, 1981; Lafère et al., 2019; Todnem et al., 1989; Dolmierski et al., 1988; Tikkinen et al., 2016; Grønning and Aarli, 2011), cognitive workload (Todnem et al., 1989; Dolmierski et al., 1988; Ergen et al., 2017; Stanley and Scott, 1995), and/or the impact of emotions/anxiety (Zec et al., 2023; Tsai et al., 2020; Boucher et al., 2022)], such impairment has not always been consistent across studies. One explanation for this could be that the cognitive skills that are typically assessed underwater (simple or lower-order cognitive processes) are less sensitive to changes in diver depth than performance-related (more complex or higher-order) cognitive skills (such as planning behaviors) (Dalecki et al., 1985).

### 4.2 Psychophysiological testing

Often supplementing neurocognitive assessments, researchers utilize psychophysiological methodologies in an attempt to identify non-invasive neurocognitive biomarkers of cognitive/behavioral deficits that may occur during diving operations. Cognitive state estimation is a well-established field that relies on a combination of vital signs and brain activity measurements (or their manifestations). While there have been one-off attempts (Phillips et al., 2022), to our knowledge, there are no fully operationalized cognitive state monitoring technologies for divers. Moreover, identification of these metrics proves to be very important to be able to monitor cognition in real-time alongside physiological states. For instance, some studies have collected EEG recordings from divers and have identified changes in the frequency domain–specifically increases in theta power and decreases in alpha power (Todnem et al., 1989; Dolmierski et al., 1988). These neural signatures empirically demonstrate utility for non-invasively monitoring a diver’s cognitive load (Stipacek et al., 2003; Käthner et al., 2014). Researchers have also explored the utility of collecting electrodermal recordings (Posada-Quintero et al., 2021) or saliva samples (Zec et al., 2023; Boucher et al., 2022) from divers as a method to assess diver stress. Interestingly, several studies have attempted to assess a diver’s cognitive state using the critical flicker fusion frequency (CFFF) (Williamson et al., 1989; Lafère et al., 2019; Tikkinen et al., 2016; Piispanen et al., 2021). The CFFF assesses the frequency that serially presented light stimuli need to be presented for an individual to perceive the series of stimuli as a steady, single light source. The CFFF appears to be modulated as a function of diver depth or interaction with gas mixtures and has been used to inform researchers of the diver’s state of arousal. Refining the types of metrics collected from a diver, enhancing analytical approaches to establish benchmarks of safe or desirable cognition, and validating that these biomarkers index the intended aspects of cognition is a clear path forward for the development of non-invasive cognitive sensing technologies.

### 4.3 Longitudinal neuroimaging and clinical diagnoses

Studies have alluded to the possibility of long-term changes in cognition and the nervous system as a function of diving (Todnem et al., 1991; Slosman et al., 2004; Balestra and Germonpré, 2016; Coco et al., 2019; Bast-Pettersen et al., 2015; Hemelryck et al., 2014); however, it is important to state that the evidence is not conclusive. More specifically, although studies investigating changes in neural signaling (Todnem et al., 1989; Todnem et al., 1991) or structural changes in brain white matter (Balestra and Germonpré, 2016; Coco et al., 2019) observe cognitive abnormalities (compared to individuals with less or no diving history), methods investigating changes in cognitive ability (i.e., perceptual, motor, spatial, attention, executive functioning, memory) vary from study to study depending on the sample and how cognition was assessed. Although there is a trend in findings suggesting decrements in several domains of memory function (Boucher et al., 2022; Williamson et al., 1989; Hobbs et al., 2014; Brookhuis et al., 1994; Todnem et al., 1991; Slosman et al., 2004; Balestra and Germonpré, 2016; Coco et al., 2019; Stanley and Scott, 1995; Bast-Pettersen et al., 2015; Hemelryck et al., 2014; Selkirk et al., 2010), other aspects of cognition are not consistently decremented. This suggests that they either do not deteriorate abnormally over time or may require more sensitive or controlled testing approaches to detect (which is difficult to achieve in a diving use case). For example, factors such as the temperature of the water during dives [however, see (Baddeley et al., 1975)], the diving depth, and the frequency of diving behaviors each may have the potential to influence the incidence rate of long-term negative neurofunctional effects (Slosman et al., 2004).

## 5 Discussion

Due to current underwater technology constraints, divers and dive supervisors have little visibility into emerging dangers before they occur and medical professionals cannot assess a diver’s condition until they return to the surface. This lack of insight limits the information available to prevent and treat diving mishaps. The development of diving devices to support real-time underwater physiological monitoring has the potential to reduce the risks associated with diving and significantly enhance diver safety and health outcomes. This paper presents common diving disorders and metrics that would be useful for a diving physiological device to enhance diver safety, but further research and testing are needed to validate the utility and feasibility of physiological metrics enhancing diver safety and health outcomes.

The diving disorder incidence data in both the recreational and military contexts demonstrates that there is an operational need to monitor divers in near-real time. The most prevalent diving disorders and causes of fatalities in the recreational context are associated with cardiac events, DCS-related symptoms, and AGEs. Although military diving environments are more controlled, with fewer mishaps due to rigorous training and supervision, the occurrence of DCS and AGE are still very prevalent.

The description of the diving disorders, and the most promising biometric data to detect them, reveals the potential opportunity for a device to be developed to track impending diving disorders in near real-time, alert the divers and their supervisors to emerging risks, enable an immediate intervention, and support the post event diving disorder analysis. Table 4 maps the most pertinent diving disorders to a measurable physiological parameter that could be incorporated into a device or other dive-able system. These relevant physiological metrics include HR, HRV, core temperature, BP, oxygen saturation (SpO_2_, SmO_2_, and StO_2_), and RR. For all of these metrics, more research is needed to validate their use in the diving context.

Despite the potential utility of measuring diver physiological metrics during a dive, few technologies have transitioned to underwater use. For instance, traditional heart rate measurement methods like ECG struggle with signal quality degradation in seawater. Similarly, although measurements like SpO_2_ would be critical for hypoxia assessment, PPG sensor effectiveness underwater can be compromised by environmental factors and require more precise calibration methods. Furthermore, the development and integration of technologies that detect gas bubbles in the blood or conditions that prognosticate bubble formation could revolutionize diver safety by providing immediate feedback on bubble presence, going beyond existing post dive technologies mentioned in Section 3.1. Although this could help mitigate the risks associated with decompression and decompression sickness, bubble detecting technologies require additional development to adapt them for an underwater use case.

There may also be benefits in developing monitoring devices for cognitive health in an underwater environment. Neurocognitive assessments, while insightful, often require pre- and post-dive evaluations and may be too invasive for near real-time application. However, methods such as EEG and electrodermal activity offer promising non-invasive biomarkers for detecting cognitive and stress-related changes during dives, and could provide additional biometric data for the diver and dive supervisor. However, as with the physiological monitoring biometrics, there needs to be significant research to validate the ability for these biometrics to be incorporated into comprehensive, non-invasive monitoring devices to enhance diver safety. Such advancements could help prevent diving mishaps, and mark a significant leap forward in the field of diving safety and survivability.
